# Unpacking Preterm Birth: How Granular Epidemiologic Analyses Can Inform Interventions

**DOI:** 10.1111/ppe.70095

**Published:** 2025-11-15

**Authors:** Daria Murosko, Kristan Scott, Diana Montoya-Williams

**Affiliations:** 1 Division of Neonatology, Children’s Hospital of Philadelphia, Philadelphia, Pennsylvania, USA; 2 CHOP PolicyLab, Philadelphia, Pennsylvania, USA

Despite decades of research, the complex mechanisms underlying preterm birth remain incompletely characterised. In addition, racial disparities in preterm birth persist both for preterm overall and when preterm birth is subclassified as either spontaneous or clinician-initiated [1]. Prior research has also established that individuals with a history of preterm birth face elevated risks of subsequent preterm delivery [2]. However, the recent analysis by Chehal and colleagues [3] in this issue of *Paediatric and Perinatal Epidemiology*, reports how factors of race, first birth gestational age, and preterm birth subtype jointly shape the risk and severity of preterm birth recurrence.

Using a population-based birth cohort from Georgia linked to hospital discharge records, Chehal et al. [3] determined the proportions of *second birth* gestational age category, stratified by both maternal race and gestational age at delivery of the *first birth*. These data clearly demonstrate the association of prior preterm birth with subsequent risk, as well as the persistent disparity in preterm births to Black women regardless of the gestational age of their first birth. However, the models across the combined race-obstetric history groupings revealed a further key finding: even when Black women had a full-term first birth (at ≥ 39 weeks), they faced an increased risk of earlier, more severe prematurity (risk ratio [RR] 2.03, 95% confidence interval [CI] [1.70, 2.43] for birth < 32 weeks; RR 1.65, 95% CI 1.53, 1.78 for birth 32–36 weeks) in their subsequent pregnancy. A similar progression was seen for Black women who first delivered at early term (37 weeks). This pattern indicates a stepwise, within-person process that elevates preterm birth risk over relatively short reproductive intervals. Furthermore, this study shows that the intersection of race and obstetric history further compounds preterm birth risks. Among Black women who delivered their first pregnancy at < 32 weeks, the rate of second preterm birth overall exceeded 33%, with one-third of those preterm births occurring < 32 weeks of gestation.

The findings of this study are important for several reasons. First, while it is well established that the pathophysiologic processes underpinning preterm birth vary by gestational age, many studies continue to analyse preterm birth as a monolithic outcome, defined solely by gestational length < 37 weeks. This is despite the fact that extremely preterm birth (< 28 weeks) is a very different outcome than late preterm birth (34–36 weeks). By designing their study around a more specific outcome variable, Chehal’s group was able to assess the relative risk of delivering a second birth at specified gestational age categories (< 32, 32–36, 37–38 weeks), each compared to a gestation of 39 weeks or more. From an analytical perspective, this choice improves statistical efficiency by avoiding multiple comparisons that would have occurred if they had modelled each second birth gestational length category separately. Although pairwise comparisons between categories that would have enabled between-group inferences were not conducted, their design facilitates a more specific epidemiologic identification of the outcome. This represents an improvement over prior research that has grouped all preterm births together. Moving forward, future work could better align with more clinically significant categories of preterm birth severity. For example, the *extremely* preterm subgroup of infants born under 28 weeks is unique, given their high risk of morbidity and mortality, though in this paper, these infants are grouped with the *very preterm* infants typically defined as infants born between 28 and 32 weeks. Granularity is important not only for understanding causal mechanisms but also for risk identification. Prevention of the high-severity, low-occurrence outcome of extremely preterm birth may require addressing different risk factors and pathways than those associated with the lower-severity but higher-occurrence outcomes of very, moderate, or late preterm birth, respectively. In short, each category of preterm birth not only has different public health implications but also perhaps distinct associated prevention strategies.

Chehal’s study is also critical because, as the authors note, their findings further support the weathering hypothesis, which links chronic exposure to systemic racism and discrimination to accelerated aging, allostatic physiologic load and earlier onset of adverse health conditions including preterm birth [4]. While weathering has traditionally been explored through cross-sectional comparisons across age groups, the longitudinal linkage across pregnancies in this analysis uniquely demonstrates how risk for preterm birth increases over time *within* a non-Hispanic Black individual’s reproductive trajectory, even after controlling for age and other medical comorbidities. Furthermore, the pattern persisted in the sensitivity analyses limited to spontaneous births conducted by this team. While allostatic load has been linked to the risk of pregnancy-related hypertensive disorders that then precipitate a clinician-initiated preterm birth [5], Chehal and the group’s work contributes to a growing body of literature suggesting that rapid accumulation of allostatic load may also contribute to the pathogenesis of the distinct outcome of spontaneous preterm birth.

Additionally, it is known that these ‘weathering forces’ vary across communities and may be impacted not only by interpersonal discrimination but by contextual risk factors [6]. Chehal et al.’s findings are a potent reminder that the different biological mechanisms that proximately result in preterm birth disparities stem from upstream, discriminatory social structures. Indeed, the authors themselves recognise that available individual-level data are too limited to capture all confounders or causal factors and that race is a ‘social construct’ that proxies for larger forces. We further commend the authors for acknowledging Georgia’s history and its link to the contemporary inequitable spatial distribution of health factors, which contributes to preterm birth disparities in Georgia.

We must, however, move beyond the descriptive epidemiological framework that relies solely on individual race as a proxy for both individual and structural factors. Querying which factor, individual race, may serve as a proxy for at the institutional level, such as healthcare, housing, education, etc., may allow for more solutions-oriented explorations. Structural factors can be directly analysed, for example, by incorporating measures of racial segregation such as redlining or state-level variation in health insurance coverage. As noted by the authors, during the study period, postpartum Medicaid coverage was extended for only 6 weeks. However, since the American Rescue Act of 2021, Medicaid postpartum coverage has been extended to 12 months in Georgia and most other states. Conversely, Georgia still has not adopted Medicaid expansion, which has been tied to decreased rates of preterm birth [7]. With a strong evidence base, such policies may be presented to legislative policymakers as structural risk or protective factors for preterm birth.

Similarly, differences in access to maternal care environments before conception and variation in healthcare practices and hospital environments during the pregnancy likely contribute to heterogeneity in preterm birth risk. To document this, however, researchers would have to adjust for the delivery hospital in their analyses. Such multi-level modelling can help uncover the upstream hospital-level factors capable of mitigating cumulative stress burdens across the reproductive life course. These types of analyses may be more informative for hospital administration policymakers than legislative policymakers, but are solutions-oriented just the same.

Chehal’s group’s work especially underscores the critical role of interpregnancy healthcare coverage and care receipt for women, particularly Black women, who have experienced preterm birth. Individuals who deliver preterm are less likely to receive recommended postpartum care, even as they have a greater prevalence of conditions such as hypertension and gestational diabetes that predispose them to recurrent preterm birth [8]. But substantial structural barriers limit access to interpregnancy care for all low-income birthing people, not just among those whose infants are born preterm. For the high proportion of women in the US who rely on Medicaid for pregnancy and postpartum healthcare coverage, for instance, recent federal changes to Medicaid mean a sizable proportion of American women risk losing healthcare coverage in those vulnerable months after birth [9].

Reframing postpartum care as preterm birth recurrence prevention can help shift the focus from reactive, pregnancy-dependent interventions (e.g., vaginal progesterone, cerclage) to preventative health policy-based interventions. One strategy is to capitalise on infant-focused healthcare interactions (i.e., neonatal hospitalisations or paediatric primary care visits) as opportunities to optimise maternal health before future conception. For example, early evidence suggests that integrating maternal care within the neonatal intensive care unit (NICU) where a (potentially) first preterm infant is being cared for may improve receipt of blood pressure screening, mental health screening and desired contraception among NICU mothers [10].

Ultimately, although documenting persistent racial inequities in preterm birth can feel redundant, critical insights can be achieved through careful interrogations of ever more granular understandings of risk profiles, as exemplified by this study. But epidemiologic analyses and policy innovation can and should go hand in hand. Findings like those in this paper become most impactful when they either inform policy or programmatic changes to close persistent racial gaps or guide future researchers on how to evaluate implemented policies or programs. Because while racial health disparities may appear intractable, they are neither inevitable nor acceptable. Evidence-informed policy change on many levels can be both the treatment for and prevention against our country’s recalcitrant racial inequities *if* we craft our epidemiologic analyses to that end.

## Data Availability

Data sharing not applicable to this article as no datasets were generated or analysed during the current study.
